# Malaysia’s First Transplanted Case of Chronic Granulomatous Disease: The Journey of Overcoming Obstacles 

**DOI:** 10.3390/children3020009

**Published:** 2016-05-17

**Authors:** Intan Hakimah Ismail, Faizah Mohamed Jamli, Ida Shahnaz Othman, Lokman Mohd Noh, Amir Hamzah Abdul Latiff

**Affiliations:** 1 Department of Paediatrics, Clinical Immunology University, Faculty of Medicine and Health Sciences, Universiti Putra Malaysia, Serdang, 43400 Selangor, Malaysia; 2 Department of Paediatrics, Serdang Hospital, Kajang, 43000 Selangor, Malaysia; idafaizah@yahoo.co.uk; 3 Paediatric Haematology, Oncology and Stem Cell Transplant Unit, Institute of Paediatrics, Kuala Lumpur Hospital, 50586 Kuala Lumpur, Malaysia; idashahnaz@gmail.com; 4 Department of Paediatrics, Universiti Kebangsaan Malaysia Medical Centre, 56000 Kuala Lumpur, Malaysia; lokman.m.noh@gmail.com; 5 Pantai Hospital Kuala Lumpur, 59100 Kuala Lumpur, Malaysia; amirlatiff@gmail.com

**Keywords:** bone marrow transplantation, clinical immunologist, chronic granulomatous disease, primary immunodeficiency, specialized laboratory facilities

## Abstract

The awareness of primary immunodeficiency (PID) in Malaysia is still not forthcoming. Certain practical issues such as lack of clinical immunologists and specialized laboratory diagnostic facilities remain to be addressed. However, great efforts taken by passionate clinicians and scientists in the immunology networking have ascertained some prevalence. Despite the limitation, all suspected cases of PID are being properly investigated and competently managed. In this case report we highlighted the obstacles we faced in managing PID patients, particularly preparing for bone marrow transplant. This is the first transplanted case of chronic granulomatous disease in Malaysia, which emphasizes the importance of collaborative work to ensure further morbidities or mortalities are prevented.

## 1. Introduction

Primary immunodeficiencies (PID) are a heterogeneous group of inherited disorders caused by defects of different components of the immune system. To date, more than 200 different spectrums of PID have been described [1] and approximately 150–180 genes have been identified [1,2]. Although the actual incidence of PID worldwide is not known, its prevalence is estimated to be between 1:500 and 1:500,000 (depending on diagnostic skills and medical resources available in the country), and it is probably higher than currently realized [3]. Recent advances in diagnostic laboratory techniques have facilitated the molecular identification and classification of the disease. Unfortunately, in Malaysia, lack of awareness in recognizing different clinical manifestations leads to the majority of PID patients remaining undiagnosed or diagnosed too late with severe morbidities. Although all signs point to potential health catastrophes, very little has been done to highlight this problem, hence diagnosis of PID is often delayed, optimum treatment missed, and patients are only referred when they have suffered critical events [4]. Research on PID has not been easy, as diagnostic skills and medical resources are scarce. Exploring the possibility of developing a training program on clinical immunology is not feasible, as it is not recognized as a distinct medical subspecialty, hence it has failed to attract pediatricians or physicians to further specialize in this area. Here, we describe the first bone marrow transplantation (BMT) for a chronic granulomatous disease (CGD) patient in Malaysia, and obstacles encountered in the management, including the larger issues of having the clinical immunology subspecialty recognized, and the necessity to train subspecialists in the field. 

## 2. Clinical Vignette 

A 3-month-old Melanau boy, born at term gestation, was admitted to a local hospital with a 3-day history of high grade fever, cough, loose stool and runny nose. Physical examination revealed a well-thriving infant with no dysmorphic facies, presence of 4 cm hepatomegaly and 1 cm splenomegaly, and absence of skin lesion or lymphadenopathy. He was the eldest of two children of non-consanguineous parents. There was no family history of PID. He was initially treated for bronchopneumonia, but the fever persisted for a few weeks despite multiple antibiotics. Retroviral, connective tissue, tuberculosis (TB) and septic screens were negative. Preliminary immunologic tests showed mild increased CD3^+^, CD4^+^ and CD8^+^ cell counts, with normal immunoglobulin (Ig)G, IgA and IgM levels for his age. Abdominal ultrasound showed hepatomegaly, while echocardiogram was normal. Multiple abscesses and granulomas in the liver and spleen, as well as multifocal segmental pneumonia, necrosis and abscesses in the lungs were seen on thoracoabdominal computerized tomography. Subsequent serial chest radiographs (CXR) showed worsening bilateral perihilar haziness. Anti-TB (isoniazid, rifampicin and pyrazinamide) was commenced for nine months, with improvement of symptoms. Repeat CXR and abdominal ultrasound after completion of treatment showed normal findings. Possibility of PID was not entertained, and no further immunological tests were requested.

At the age of 18 months (six months after the completed anti-TB), he presented again with aone-week history of intermittent fever and painful bilateral cervical and submandibular lymph nodes. TB workup was negative. Bone marrow aspiration did not suggest malignancy. Excision biopsy of submandibular lymph nodes revealed suppurative adenitis on histopathologic examination. The biopsy site was noted to have healed poorly, which raised the possibility of reactivation of TB, and consequently anti-TB was restarted, but with no improvement. Five weeks later, indirect fluorescent antibody for melioidosis showed a titer of 1:1640. He was then treated with augmentin, doxycycline and co-trimoxazole, while anti-TB was discontinued after two months. Because of recurrent deep-seated infections, he was referred to our center for probable PID. The second immunologic investigations revealed IgG 1030 mg/dL (normal range for age 500–1000 mg/dL), IgA 132 mg/dL (normal range for age 40–80 mg/dL), and IgM 130 mg/dL (normal range for age 35–75 mg/dL). Lymphocyte subsets showed normal T and B cells for his age. Chemiluminescence measurement of respiratory burst was more than 95% depressed. Molecular genetic studies identified autosomal recessive mutations in *Neutrophil Cytosolic Factor 1* (*NCF1*) gene (two nucleotide deletion at the beginning of exon two) resulting in lack of p47phox protein expression. Thus, almost a year later, the patient was diagnosed to have autosomal recessive CGD. He was discharged home with prophylactic cotrimoxazole and itraconazole, with no further major infections. Subsequently, he was referred to the BMT team. Due to unforeseen circumstances and administrative issues, haematopoietic stem cell transplantation (HSCT) was only carried out five years after diagnosis of CGD was made. Human leukocyte antigen (HLA) typing from his younger brother was a complete match for HLA class I (HLA-A: 11, 24; HLA-B: 07, 75; HLA-Cw: 08) and class II (HLA-DR: 12, 15; HLA-DQ: 05, 07). Myeloablative conditioning consisted of busulphan and cyclophosphamide. Graft-versus-host disease prophylaxes were methotrexate and cyclosporine. Stem cell source contained 8.34 × 10^8^/kg nucleated cells, 8.9 × 10^6^/kg CD34^+^ cells and 6.3 × 10^6^/kg CD3^+^ cells. The peri-transplant period was uncomplicated, while post-transplant complications were mild but expected—febrile neutropenia and mild mucositis. His superoxide production normalized two months after transplantation. 

## 3. Discussion

Malaysia has a population of 29.6 million (statistic 2013), and about 10% of the citizens are children under 5 years of age. The first reported case of PID in Malaysia dates back to 1977 [5], followed by scattered case reports describing clinical features with limited immunological data [6,7,8,9,10,11,12,13,14]. PID had been diagnosed and recorded in Malaysia since 1986 at a modest rate of 2.5 per-year until 2002, and 3 per-year until 2006 by a single pediatric immunologist (LMN). From 2007 until 2011, with an additional clinical immunologist (AHAL), the number recorded increased (average of 24 cases per-year) almost 10 times (Proceeding of the 15th European Society for Immunodeficiencies Meeting, 3–6 October 2012, Florence, Italy). Prior to 2007, basic immunological tests were not routinely done, while molecular tests were sent overseas due to a lack of clinical immunologists and facilities within the country. In Malaysia, the average interval from the onset of first symptoms appeared to the age of diagnosis made (diagnostic delay) was 3.87 years [4].

Under the initiation of LMN and AHAL, the Clinical Immunology Unit at Universiti Putra Malaysia and Cluster of Immunological Science at Universiti Sains Malaysia were developed to provide specialist care service and diagnostic immunology tests for children with PID. Furthermore, the Institute for Medical Research (IMR) Malaysia has embarked on providing specialized immunological tests, including limited molecular genetic tests, which are being further expanded. This collaborative networking has resulted in more cases being properly diagnosed and managed.

Although clinical immunology is not included in the national specialist registry, a group of dedicated clinical immunologists, clinicians and scientists from both Ministry of Health and Universities, under the umbrella of Malaysian Primary Immunodeficiencies Network (MyPIN) and Malaysian Society of Allergy and Immunology (MSAI), have conducted a series of symposiums, such as the National Clinical Immunology Symposium (NACLIS), in order to increase awareness of PID amongst the medical fraternity that encompasses both clinical and scientific branches. Also, the Malaysian Patient Organization for Primary Immunodeficiencies (MyPOPI) was recently established as a platform for parents and patients to organize activities for generating awareness within the community. 

CGD is an inherited phagocytic disorder resulting from failure of neutrophils to produce oxidases to kill defined-spectrum organisms [15]. Most patients are diagnosed in early childhood when they present with recurrent, life-threatening, deep-seated bacterial and fungal infections and granuloma formation [15]. The prevalence is 1 in 120,000 live births [15], although true incidence could be higher due to under-diagnosis of milder phenotypes. In Malaysia, until 2006, the frequency of CGD was reported at 7.6% (4/52), and delay in diagnosis was 4.73 years [4]. Our case contributes to the existing four cases of CGD reported in Malaysia until 2006 [10,16]. The standard care for CGD patients is antibiotic and antifungal prophylaxes. Allogeneic HSCT is currently the only curative therapy with research on stem cell gene therapy that is underway [15].

BMT in Malaysia was first conducted in 1987, with a total of 1518 transplantations being performed up to 2008. The majority were allogeneic and usually offered to patients with leukemia, thalassemia major and aplastic anemia [17]. Mortalities post-transplantation are usually caused by underlying disease or infection-related complications [18]. However, for patients with PID, facilities for HSCT are not readily available; the majority of patients usually succumb to death even before BMT is initiated or considered. In reviews of BMT Registry for PID [19,20], the majority of patients are less than 10 years old at the time of transplant. Our patient was transplanted at age six years, and he was discharged home 19 days after transplant, without major complications. The most important observation in this report is that PID is now an emerging disease in Malaysia, not because it is a new disease entity, but rather because more cases are being diagnosed as a result of increasing awareness and better diagnostic facilities [4]. Equally, BMT should be contemplated as a curative option once diagnosis is confirmed, preferably before the onset of organ damage and risk of serious transplant complications increases.

## 4. Conclusions 

Early diagnosis and prompt treatment are keys for successful outcome of PID. The emphasis is that Malaysia is capable of managing PID, but requires greater infrastructure and training opportunities to be comparable with management elsewhere. As long as Clinical Immunology is regarded as an “orphan” subspecialty, this entire vision cannot be achieved.

## Abbreviations

The following abbreviations are used in this manuscript:

CGD	Chronic Granulomatous Disease
BMT	Bone marrow transplantation
PID	Primary Immunodeficiencies
HSCT	Haematopoietic stem cell transplantation
